# Authors’ reply to comments on erector spinae plane block versus thoracic paravertebral block in pediatric percutaneous nephrolithotomy

**DOI:** 10.1016/j.bjane.2026.844818

**Published:** 2026-08-13

**Authors:** Fatma Nabil, Ahmed M. Mandour, Amr M. Abdelgawad, Deiaaeldin M. Tamer, Ahmed A. Shahat, Mohamed Anwar, Hany M. Osman

**Affiliations:** a Assiut University, Faculty of Medicine, Department of Anesthesia, Intensive Care, and Pain Management, Assiut, Egypt; b Assiut University, Faculty of Medicine, Department of Urology, Assiut, Egypt; c Assiut University, Faculty of Medicine, Pediatric Department, Assiut, Egypt

*Dear Editor*,

We thank Dr. Peng and Dr. Li,^1^ and Dr. Padhi and colleagues^2^ for their thoughtful Letters to the Editor regarding our article on erector spinae plane block versus thoracic paravertebral block in pediatric patients undergoing percutaneous nephrolithotomy.^3^ Their comments provide an important opportunity to clarify the interpretation of our findings, particularly regarding trial design, analytic population, procedural time, and safety considerations.

First, our study was designed and powered for the primary outcome as a superiority trial, not as an equivalence or non-inferiority trial. Therefore, we agree that the absence of statistically significant difference in the primary outcome should not be interpreted as evidence of equivalence or non-inferiority between ESPB and thoracic PVB. With regard to the conclusion statement “could be considered as an easy alternative”, we intended the practical and clinical perspective related to the comparable analgesic effects rather than claiming therapeutic equivalence or superiority.

With regard to the methodological concerns raised by Padhi et al., because the excluded patient did not undergo the planned PCNL procedure after randomization, an intention-to-treat sensitivity analysis including this participant was not considered clinically meaningful. All randomized patients who received the allocated intervention and completed the planned PCNL procedure were included in the final analysis. The intended and actually administered fentanyl dose was 1µg.kg^-1^. The previous notation as 1 mg.kg^-1^ was a unit error.

We appreciate the important point of Peng and Li that a 40-second difference in the time to conduct the blocks is of negligible clinical benefit despite being statistically significant and we had already stated in the discussion that this result, as a secondary outcome, should be interpreted cautiously. However, it is worth re-emphasizing that all the blocks were conducted by a single anesthesiologist who has a substantial experience in regional anesthesia for pediatric patients. Although this was not specifically evaluated in the current study, the shorter performance time of ESPB may be more relevant in settings where clinicians are less experienced with thoracic PVB or where obtaining the paravertebral sono-anatomic view is more challenging as greater caution is required to avoid plueral puncture.

With regard to the concern raised by Padhi et al. we agree that the current evidence comparing erector spinae plane block and paravertebral block remains inconsistent across different surgical settings. Therefore, we intended to design our methodology to be limited to one surgical procedure in the pediatric population. Hence, our conclusion is confined to this context.

Regarding safety, we agree with Peng and Li that the safety profile in the pediatric population has more robust evidence for PVB since it is an older technique, and we also agree that the current study was not powered to detect the complications as we already mentioned that in the manuscript as a limitation. As a relatively new regional analgesic technique, promising data have started to emerge supporting the safety of ESPB in the pediatric population.^4^ However, larger studies are still required to evaluate rare adverse events.

In conclusion, we appreciate the observations raised by the correspondents. We agree that our findings should be interpreted in the context of a superiority trial, and we recommend larger powered studies to evaluate the safety of ESPB in pediatric patients.

## Conflicts of interest

The authors declare no conflicts of interest.

## Data Availability

No new data were generated or analyzed in this article. Data sharing is not applicable to this article.
